# Drugs Targeting p53 Mutations with FDA Approval and in Clinical Trials

**DOI:** 10.3390/cancers15020429

**Published:** 2023-01-09

**Authors:** Shigeto Nishikawa, Tomoo Iwakuma

**Affiliations:** 1 Department of Pediatrics, Division of Hematology & Oncology, Children’s Mercy Research Institute, Kansas City, MO 64108, USA; 2 Department of Cancer Biology, University of Kansas Medical Center, Kansas City, KS 66160, USA

**Keywords:** mutant p53, clinical trial, reactivation, synthetic lethality, vulnerability

## Abstract

**Simple Summary:**

Mutations in the tumor suppressor p53 (p53) occur in ~50% of human cancers, the majority of which are missense mutations. Mutations in p53 not only impair the tumor suppressive function, but also confer missense mutant p53 (mutp53) with oncogenic activities independent of wild-type p53 (wtp53). Since p53 mutations are cancer-specific, several approaches targeting them have been taken to develop novel cancer therapies, including restoration or stabilization of wtp53 conformation from mutp53, rescue of p53 nonsense mutations, depletion of mutp53 proteins, and induction of p53 synthetic lethality or targeting of vulnerabilities imposed by p53 deficiencies (activated retrotransposons) or mutations (enhanced YAP/TAZ). Here, we summarize clinically available investigational and FDA-approved drugs that target p53 mutations for their mechanisms of action and activities to suppress cancer progression.

**Abstract:**

Mutations in the tumor suppressor p53 (p53) promote cancer progression. This is mainly due to loss of function (LOS) as a tumor suppressor, dominant-negative (DN) activities of missense mutant p53 (mutp53) over wild-type p53 (wtp53), and wtp53-independent oncogenic activities of missense mutp53 by interacting with other tumor suppressors or oncogenes (gain of function: GOF). Since p53 mutations occur in ~50% of human cancers and rarely occur in normal tissues, p53 mutations are cancer-specific and ideal therapeutic targets. Approaches to target p53 mutations include (1) restoration or stabilization of wtp53 conformation from missense mutp53, (2) rescue of p53 nonsense mutations, (3) depletion or degradation of mutp53 proteins, and (4) induction of p53 synthetic lethality or targeting of vulnerabilities imposed by p53 mutations (enhanced YAP/TAZ activities) or deletions (hyperactivated retrotransposons). This review article focuses on clinically available FDA-approved drugs and drugs in clinical trials that target p53 mutations and summarizes their mechanisms of action and activities to suppress cancer progression.

## 1. Introduction

The tumor suppressor p53 was initially thought to be an oncogene since the originally cloned cDNA contained a missense mutation [1]. Indeed, mutant p53 (mutp53) functions as an oncogene independent of wild-type p53 (wtp53) [2,3]. Later, however, in studies using p53 cDNA without any mutations, the wtp53 was proven to be a bona fide tumor suppressor [4,5].

**Figure 1:**
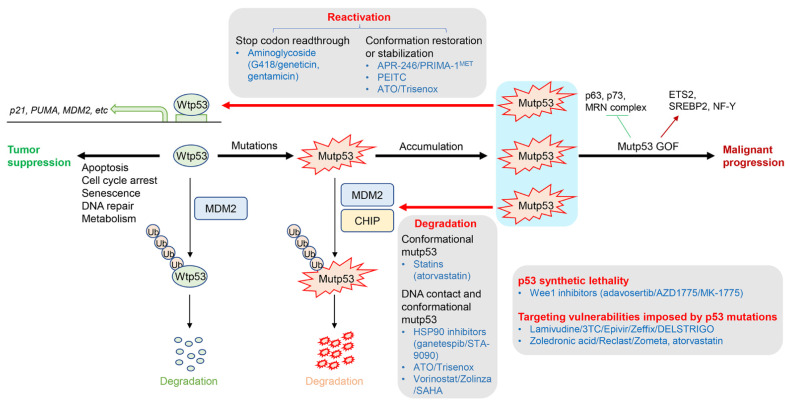
Function of wtp53, mutp53 GOF, and strategies to target p53 mutations.

p53 is a transcription factor and regulates the transcription of numerous downstream target genes involved in apoptosis, cell cycle arrest, senescence, DNA repair, and cellular metabolism, thereby functioning as a tumor suppressor [6]. The protein level and activity of wtp53 remain low in non-stressed conditions mainly through degradation by MDM2 [7]. Under genotoxic conditions, wtp53 is stabilized and activated through post-translational modifications (PTMs) by phosphorylation or acetylation to induce cell cycle arrest and/or cell death (Figure 1). Once the wtp53 function is impaired due to mutations or deletions, cells lose control of their growth, which promotes tumorigenesis [2,3].

Accumulating evidence indicates that p53 is the most frequently mutated gene in human cancers, with mutations in over 50% of human cancers [8]. The majority of p53 mutations are missense mutations in the DNA-binding domain. Mutations in p53 result in the loss of function (LOS) as a transcription factor and a tumor suppressor. However, missense mutp53 frequently accumulates in tumors to promote malignant progression, metastasis, and drug resistance in a manner independent of wtp53. These oncogenic mutp53 activities are referred to as gain of function (GOF) (Figure 1). The mechanism of mutp53 GOF is mainly caused by mutp53’s ability to bind to tumor suppressors (e.g., p63, p73, MRN complex) and oncogenes (e.g., ETS2, SREBP2, NF-Y) to alter the functions of these binding partners [2,3,9]. Clinically, the presence of mutp53 in tumors is well correlated with advanced clinical stages, metastases, and poor outcomes in patients with multiple types of cancer [10,11]. Given that mutations in p53 are generally observed specifically in tumors and are rare in non-tumor tissues, mutp53 is an ideal therapeutic target for cancer therapy.

Several strategies to target p53 mutations have been taken (Figure 1). The first strategy is to directly target missense mutp53 to restore the activity of wtp53 or stabilize the wtp53 conformation. Drugs or compounds that have this function are referred to as reactivators. The most representative drug in this group is APR-246 (eprenetapopt/PRIMA-1^MET^), which is in several phase 2 or 3 clinical trials. Although the tumor suppressive effects of APR-246 in mutp53-carrying tumors in mouse models have been shown to be successful, it is not yet approved by the Food and Drug Administration (FDA) [12]. The second strategy is to induce degradation or depletion of missense mutp53, which capitalizes upon the addiction of cancer cells to mutp53 and potentially restores the activities of some tumor suppressors, including p63 and p73, whose functions have been suppressed by mutp53. Drugs or compounds employed for this strategy include HSP90 inhibitors or statins, cholesterol-lowering drugs that are shown to induce degradation of mutp53, leading to tumor suppression [13,14]. The third strategy is to induce cell death specifically in cancer cells with p53 deletions or mutations, so called p53 synthetic lethality [15]. This strategy often targets vulnerabilities imposed by p53 deficiency or mutp53 GOF, instead of directly targeting mutp53. Drugs or compounds used for this strategy include Wee1 inhibitors or inhibitors of DNA damage response signaling (e.g., ATR or Chk1/2 inhibitors) [16,17,18]. Other strategies include wtp53 rescue of non-sense mutations in p53 [19,20], inhibition of retrotransposon activated upon p53 deficiency [19], and inhibition of YAP/TAZ function activated by mutp53 [21,22]. Here, we have summarized updated information about drugs developed for the aforementioned strategies that are approved by the FDA or are in clinical trials, including their mechanisms of action and activities to suppress cancer progression (Table 1).

## 2. Drugs Targeting p53 Mutations

### 2.1. Restoration or Stabilization of wtp53 Conformation from Missense mutp53

Most mutp53 lose the activity of wtp53 as a transcription factor and a tumor suppressor. However, accumulated evidence indicates that the tumor suppressive activity can be restored under specific conditions including temperature shift, exposure to synthetic peptides from 53BP2-derived “CDB3” and p53 C-terminal domain “Peptide 46”, and insertion of second-site mutations or an N-terminal deletion [36,37,38]. Many investigators have attempted to discover small molecule compounds that restore the wtp53 conformation, transcriptional activity, and tumor suppressive activity [2]. For example, CP-31398 is one of the earliest mutp53-reactivating compounds that can stabilize active confirmation of p53 and promote p53-mediated tumor suppression [39,40]. Additionally, JC744, an aminobenzothiazole analog, was recently identified as a new compound that could specifically bind to and stabilize the p53Y220C mutant in vitro in the nanomolar range, through a narrow surface pocket induced by p53Y220C [41]. However, only a few compounds are in clinical trials. These include APR-246 (eprenetapopt/PRIMA-1^MET^), phenethyl isothiocyanate (PEITC), and arsenic trioxide (ATO/Trisenox).

#### 2.1.1. APR-246 (Eprenetapopt, PRIMA-1^MET^)

PRIMA-1 was identified as a small molecule compound that suppressed the growth of Saos-2 osteosarcoma cell line expressing p53R273H [12]. PRIMA-1 was shown to restore the p53’s sequence-specific DNA-binding and growth-suppressing activities in multiple cancer cell lines with different p53 mutants, including R110L, V157F, R175H, L194F, R213Q/Y234H, G245V, R248Q, R273C, R273H/P309S, R280K, and R282W. Thus, PRIMA-1 and its methylated analog PRIMA-1^MET^ (also known as eprenetapopt or APR-246) rescue the p53’s transcriptional activity from both DNA contact and structural p53 mutants [12,42]. In mouse models, APR-246 successfully inhibits tumor progression of multiple cancer cell lines, including osteosarcoma Saos2 exogenously expressing p53R273H, small cell lung cancer GLC16 (p53R273L) and DMS53 (p53S241F), and colon cancer DLD-1 (p53S241F) [12,42,43]. Additionally, Fransson et al. [44] demonstrated synergetic effects of APR-246 with DNA-damaging drugs (cisplatin, carboplatin, doxorubicin) using 10 primary ovarian cancer cells from patients. As a mechanism of action, APR-246 is converted to the biologically active methylene quinuclidinone (MQ) compound that covalently binds to cysteine residues in the core domain of mutp53 to promote refolding and restoration of wtp53’s DNA-binding activity [45]. Specifically, among 10 cysteine residues in p53 [46], two residues at C124 and C277 appear to be crucial for the APR-246-mediated functional restoration of p53R175H [47].

A phase 1b clinical trial (NCT04383938) was performed to examine the safety and efficacy of APR-246 combined with pembrolizumab, an immune checkpoint inhibitor, for patients with advanced/metastatic solid tumors [23]. Due to insufficient sample sizes (37 patients evaluated), a formal assessment of the treatment efficacy was not executed, but the combination was well tolerated and did not cause unmanageable adverse effects. An investigation comparing the therapy response between p53-mutated vs. wild-type tumors was not yet made in this study.

Two clinical trials for p53-mutated myelodysplastic syndromes (MDS)/acute myeloid leukemia (AML) with 20–30% marrow blasts (oligoblastic AML) (phase 1b/2, NCT03072043) [24] and for p53-mutated MDS/AML, including cases with more than 30% blasts (phase 2, NCT03588078) [25,48], were performed to test the safety and efficacy of APR-246 in combination with azacitidine. In the NCT03072043 trial, the response rates for MDS and oligoblastic AML were 73% and 64%, respectively [24]. In the NCT03588078 trial, the response rates for MDS and AML were 62% and 33%, respectively [25]. The combination treatment was well tolerated. In both studies, responding patients had significant reductions in the frequency of p53 variant alleles, determined by negativity of next generation sequencing (NGS). Additionally, a phase 2 trial (NCT03931291) was conducted to investigate the efficacy and safety of APR-246 in combination with azacitidine for p53-mutated MDS or AML patients as post-hematopoietic stem-cell transplantation (HCT) maintenance therapy [26]. This treatment was also well tolerated with an acceptable safety profile. Importantly, the 1-year relapse-free survival (RFS) was improved to 60%, as compared with a previous report showing a 1-year RFS of approximately 30% for p53-mutated MDS patients [49]. APR-246 is currently in a phase 3 clinical trial (NCT03745716) to investigate the possible additive effects of azacitidine with APR-246 on inhibiting the progression of p53-mutated MDS. Additional clinical trials testing the efficacy of APR-246 in combination with other drugs on high grade serous ovarian cancer (HGSOC) and myeloid malignancies have also been underway (NCT02098343, NCT03268382, NCT04214860). However, there is currently no clinical trial testing the effects of APR-246 as a single agent.

#### 2.1.2. Phenethyl Isothiocyanate (PEITC)

PEITC is extracted from cruciferous vegetables (e.g., watercress) and has been suggested to have anti-cancer effects [50]. Epidemiological studies also support the preventive effects of dietary isothiocyanates in different types of human cancers [51,52,53]. Aggarwal et al. [54] demonstrated that PEITC inhibits viable proliferation of cancer cells expressing p53R175H (Sk-Br-3, AU565) and p53R175L (HOP92) more effectively than those with wtp53, p53 null, and DNA contact mutp53 (p53R248W, p53R273H, p53R280K). As a mechanism, PEITC enhances zinc-mediated refolding of p53R175H or restoration of intact p53 structure. Moreover, PEITC induces reactive oxygen species (ROS) by impairing the GSH antioxidant system, which greatly contributes to mutp53 reactivation and inhibition of the growth of cells and tumors [54]. However, it remains unclear how PEITC restores the intact p53 structure of p53R175H or R175L in a manner dependent on zinc and whether PEITC can reactivate other zinc-binding and conformational mutp53.

A phase 2 clinical trial (NCT01790204) has been performed to examine whether PEITC-containing juice from watercress could reduce the number of oral cells having p53 mutations (not specified for p53R175H or R175L); however, the outcome of this study has not yet been reported.

#### 2.1.3. Arsenic Trioxide (ATO/Trisenox)

Arsenic trioxide (ATO, also known as Trisenox) is an FDA-approved drug to treat acute promyelocytic leukemia (APL) that is characterized by the expression of PML-RARα fusion protein [55,56]. ATO induces degradation of PML-RAR alpha, differentiation of APL cells, mitochondrial oxidative stress and apoptosis, repression of c-fos, and upregulation of p53 [55,56]. Intriguingly, ATO is shown to induce PirH2-mediated ubiquitination and degradation of both DNA contact and structural mutp53 (see Section 2.2.3) [57].

Recently, Chen et al. [58] demonstrated that ATO rescues p53 activity from structural mutp53 through promotion of p53 folding by covalently binding to multiple cysteines in p53. ATO is selected through a series of screens. These include (1) inhibition of the growth of NCI-60 cell lines carrying structural p53 mutations (p53R175H, p53R175L, p53G245S, p53R249S), (2) in silico studies to identify the ability to bind multiple cysteines, and (3) biochemical approaches to select compounds with refolding potential using a p53 conformation-specific antibody (PAb1620). ATO restores the p53’s ability to transactivate p53 downstream targets including CDKN1A, PUMA, and MDM2 in cells expressing p53R175H, p53R249S, p53G245S, and p53R282W, but not in cells expressing p53R248Q and p53R273H. In vivo mouse model studies show that ATO inhibits tumor growth of xenografts (p53R175H) and PDXs (p53R282W). Moreover, of the 25 most frequent p53 mutations covering 40.87% of p53 missense mutations, ATO rescues intact p53 structure from mutp53, except DNA contact mutants (S241F, R248L/Q/W, R273C/H/L) and some structural mutants (V157F, R158H, Y205C, Y220C) that are distant from the ATO-binding site [58]. Currently, several clinical trials to examine the effects of ATO on inhibiting p53-mutated cancers (MDS, AML, refractory solid tumors, recurrent and metastatic ovarian and endometrial cancer) are underway in China (NCT03855371, NCT04869475, NCT04489706, NCT04695223). However, the outcomes of these studies have not yet been reported.

#### 2.1.4. Aminoglycosides to Rescue p53 Nonsense Mutations

Another interesting approach targeting p53 mutations is to rescue p53 nonsense mutations and restore wtp53 activity using aminoglycosides such as clinically available gentamicin. Approximately 10% of p53 mutations are nonsense mutations that lead to premature termination of protein translation [20,59]. The resulting truncated p53 proteins are frequently non-functional or lack expression due to nonsense-mediated mRNA decay. R213X and R196X in p53 are the two most frequent nonsense p53 mutations in human tumors [20,60].

Stop codon readthrough in mammalian cells during protein translation by aminoglycoside antibiotics was first reported by Burke et al. [61] in 1985, in which suppression of an amber mutation (TAG) by aminoglycosides restored the activity of mutated chloramphenicol transferase (CAT) to ~20% of the wild-type CAT activity. Since then, several groups have experimentally demonstrated restoration of p53 activity with upregulation of the downstream target genes via stop codon readthrough [19,20,62]. Furthermore, MDM2 inhibitors (e.g., Nutlin-3a, MI-773) increase the restored full-length p53 levels, p53 transcriptional activity, and cytotoxicity by aminoglycosides (G418/geneticin, gentamicin) in HDQ-P1 cells homozygous for R213X as well as H1299 cells exogenously expressing R213X [20]. These observations strongly suggest that clinically available aminoglycosides can be used to restore p53 activity in cells or tumors with p53 nonsense mutations. However, no clinical trials with a focus on p53 nonsense mutations are underway. The lack of clinical trials may be due to several side effects caused by aminoglycosides and potential adverse effects caused by stop codon readthrough of unknown or unidentified genes with nonsense mutations.

### 2.2. Depletion or Degradation of mutp53 Protein

Increasing evidence suggests that mutp53 is inherently unstable. However, genotoxic stress, including oncogenic stress and irradiation, stabilizes mutp53 to promote its GOF activities in tumors [63]. Critically, knockdown of mutp53 reduces malignant properties of cancer cells [13,64,65,66,67,68], suggesting addiction of cancer cells to oncogenic mutp53. The exact mechanisms of mutp53 stabilization or degradation remain unclear, but there are mutp53-specific degradation mechanisms that are distinct from those for wtp53 [69]. Indeed, several compounds induce mutp53 degradation without altering wtp53 levels [13,14], and some mutp53-depleting drugs are in clinical trials. These include HSP90 inhibitors (ganetespib/STA-9090), statins (atorvastatin), ATO/Trisenox, and vorinostat/Zolinza/suberoylanilide hydroxamic acid (SAHA), as described below in detail.

#### 2.2.1. HSP90 Inhibitors (Ganetespib/STA-9090)

HSP90 is an ATPase-dependent molecular chaperone that regulates protein folding and stabilization or degradation under physiological and stress conditions, thereby controlling proteostasis [70]. HSP90 has several hundred protein substrates, so called clients, including c-Kit, EGFR, and Bcr-Abl. HSP90 is also shown to bind to both wtp53 and mutp53 [71]. HSP90 regulates the activity of wtp53 under physiological and non-physiological conditions [71,72]. HSP90 also contributes to mutp53 stabilization by inhibiting MDM2 and CHIP ubiquitin ligases by forming a complex [73,74]. Thus, the roles of HSP90 in wtp53 and GOF mutp53 activity or protein stability are diverse; the diversity is likely dependent on the cellular context, type of stress, and structure of clients.

Several HSP90 inhibitors have been developed (e.g., 17-AAG) [75,76,77,78]. Specifically, ganetespib/STA-9090, which is a synthetic resorcinolic triazolone inhibitor of HSP90, is in clinical trials. Ganetespib binds to the ATP-binding domain at the N-terminus of HSP90, which inactivates or downregulates oncogenic HSP90 clients, leading to tumor suppression [77,79]. It shows higher potency to inhibit viable cell proliferation of 57 transformed cell lines derived from both hematologic and solid tumors than 17-AAG [80]. It also inhibits in vivo growth of human tumor xenografts with activating mutations or amplifications in oncogenic factors, such as EGFR, c-Met, BCR-ABL, B-RAF, c-KIT, and HER2, in multiple types of cancer [79,80,81,82,83,84]. However, studies have not addressed the dependence of ganetespib’s effects on wtp53 and mutp53.

Alexandrova et al. [13] reported that genetic and pharmacologic depletion of mutp53 (R248Q) by SAHA (vorinostat/Zolinza, see Section 2.2.4.), 17AAG, and ganetespib leads to inhibited growth of cancer cells expressing mutp53 in a manner dependent on mutp53. These include human MDA-MB-231 (p53R280K) cells, mouse sarcoma or lymphoma cells from p53R248Q/− mice, and lymphoma cells from p53R172H/R172H mice. Similar tumor suppressive effects of 17AAG +/− SAHA are observed using MDA-MB-468 (p53R273K), T47D (p53L194F), SKBR3 (p53R175H), DU145 (p53P223L/V274F), ES2 (p53S241F), and H1975 (p53R273H) cell lines and their xenografts. Moreover, prophylactic and therapeutic treatments of p53R172H/R172H and p53R248Q/− mice with ganetespib inhibit tumor growth in these mouse models and extend their survival, which is not observed in control p53−/− mice. The in vivo drug effects are well correlated with upregulation of HSP70, a marker of HSP90 inhibition [85], as well as mutp53 reduction. Importantly, ganetespib shows minimal effects on wtp53 levels in cancer cells and xenografts [13]. These findings strongly suggest that depletion of mutp53 by HSP90 inhibition specifically suppresses growth of tumors expressing DNA contact or structural mutp53.

Many clinical trials of HSP90 inhibitors have been performed; however, most studies do not stratify the p53 status [75]. Only one clinical trial (NCT02012192) in phase 1 and 2 has an inclusion criterion of mutp53 and investigates additive effects of ganetespib to paclitaxel on p53-mutated platinum-resistant ovarian cancers. The outcome report confirms the safe use of the combination [27]. However, based on the meeting abstract of the 2018 ASCO (American Society of Clinical Oncology) Annual Meeting, which reports outcomes of the same trial using a total of 133 patients [86], the addition of ganetespib to paclitaxel does not significantly improve the survival in patients with platinum-resistant ovarian cancers. Trials for different stages of ovarian cancers or different cancer types with mutp53 may need to be considered in the future.

#### 2.2.2. Statins (Atorvastatin)

Statins are a group of drugs that lower the production of cholesterol by specifically inhibiting HMG-CoA reductase activity, a rate-limiting step of the mevalonate pathway [87]. Although statins have been implicated in inhibiting cancer progression, the clinical efficacy has been controversial [88,89,90].

Previously, our group found that several statins (lovastatin, atorvastatin, mevastatin, rosuvastatin) induce CHIP-mediated degradation of mainly structural or conformational mutp53, based on a screen of ~9000 compounds using Saos2 (p53null) cells expressing a p53R175H-luciferase fusion protein [14]. Lovastatin depletes multiple conformational p53 mutants including p53R156P (KHOS/NP), p53V157F (H-2087), p53R175H (CAL33, SKBr3), and p53Y220C (BxPC3) through a reduction in mevalonate-5-phosphate (MVP), a metabolite of the mevalonate pathway, with minimal effects on DNA contact p53 mutants, such as p53R273H (HT29) and p53R280K (MDA-MB-231), as well as wtp53. Reduced MVP inhibits binding between mutp53 and DNAJA1, a member of the HSP40 family (also known as J-domain proteins (JDPs)), which triggers degradation of mutp53 mediated by CHIP, but not MDM2, ubiquitin ligase. Atorvastatin and rosuvastatin successfully inhibit tumor growth of conformational mutp53-expressing cancer cells with reduced mutp53 levels in tumors. Atorvastatin also inhibits in vivo tumor growth of transformed MEFs expressing p53R172H, but not wtp53 or p53 null [14]. Thus, statins can be used to inhibit the malignant progression of tumors carrying structural or conformational mutp53. These findings may also suggest that clinical studies using statins need to take into consideration the status of p53 in tumors.

Similarly, Ingallina et al. [91] showed that cerivastatin depletes multiple mutp53 (R273H, R249S, L194F, M237I, R175H) with minimal effects on wtp53. They showed that cerivastatin inhibits RhoA geranylgeranylation, which somehow reduces HDAC6 activity and increases HSP90 acetylation (inactive), leading to the dissociation of HSP90 from mutp53 and subsequent mutp53 degradation by MDM2.

Despite the increasing evidence of mutp53 depletion by statins, only a few clinical reports have examined the statins’ effects on cancer progression or survival by stratifying the p53 mutation status, while the majority of clinical studies have been performed without stratifying the p53 status. For example, statin-user patients with lung adenocarcinoma had significant clinical benefits for improving overall survival compared with non-statin user patients only when tumors had p53 mutations, supporting the statins’ tumor inhibitory effects [92]. On the other hand, statin use appears to be non-beneficial for colon cancer even when patients are stratified by the p53 status [93]. The discrepancy may occur due to the differences in the dose of statins used, types of statins used, and type of p53 mutations in tumors. Indeed, Parrales et al. [14] indicated that a high dose of statins is required for inducing mutp53 degradation in tumors using mouse models.

Regarding clinical trials studying statin’s significance on cancer inhibition, some phase 3 clinical trials were performed in hepatocellular carcinoma [94], gastric cancer [95], and small cell lung cancer (SCLC) [96]; however, none of these studies considered the p53 status in tumors. Furthermore, none of these trials showed any survival benefit of statin’s addition to conventional standard chemotherapy. Recently, two clinical trials with stratification of the p53 mutation status in tumors have started. One is a phase 2 trial (NCT04767984) testing the effects of atorvastatin for patients at risk of colon cancer with longstanding ulcerative colitis who have dominant-negative missense p53 mutations. The other is a window-of-opportunity trial (NCT03560882) to determine whether atorvastatin successfully decreases the level of conformational mutp53 in solid tumor and relapsed acute myeloid leukemia. Results of these studies may further confirm that p53 status in tumors is a crucial factor in determining the efficacy of statins on tumor inhibition.

#### 2.2.3. ATO/Trisenox

As described in Section 2.1.3, ATO was recently shown to possess the ability to bind several structural mutp53 and restore the wtp53 conformation and activity [58]. However, ATO was previously suggested to deplete several conformational and DNA contact p53 mutants (p53R175H, p53H179Y/R282W, p53R248W, p53R273H) in a manner dependent on the upregulation of PirH2 ubiquitin ligase [57,97]. ATO was initially found to induce degradation of proteins with high levels of cysteine residues and vicinal thiol groups, including PML (promyelocytic leukemia protein) and PML-RARα [98,99]. Chen’s group [97] first reported that ATO accumulates wtp53 while it induces degradation of several mutp53 (p53H179Y/R282W, p53R248W, p53R273H). Later, the same group [57] showed that ATO induces PirH2-mediated ubiquitination and degradation of mutp53 in HaCaT (p53H179Y/R282W) and MIA PaCa-2 (p53R248W) cell lines. They additionally showed that 17AAG or SAHA cooperates with ATO for mutp53 depletion and inhibition of proliferation of HaCaT and MIA PaCa-2 cell lines. Based on these findings of ATO-mediated mutp53 degradation, two clinical trials are underway (phase 2: NCT03381781 for AML with p53 mutations; phase 3: NCT03377725 for MDS with p53 mutations). Since ATO appears to have dual effects on mutp53 (degradation of both DNA contact and structural mutp53 and reactivation of structural mutp53), further clinical trials examining the ATO’s dual activities need to be executed.

#### 2.2.4. Vorinostat (Zolinza/Suberoylanilide Hydroxamic Acid: SAHA)

Vorinostat/Zolinza/SAHA is an FDA-approved inhibitor of class I, II, and IV histone deacetylases and epigenetically regulates the malignant properties of multiple cancer types [100]. The first observation of mutp53 depletion by HDAC inhibitors was reported by Blagosklonny et al. [101], in which an HDAC inhibitor (HDACi), FR901228 (romidepsin, Istodax), reduced protein levels of both structural and DNA contact mutp53 (R175H, R280K, V274F/P223L) and preferentially inhibited viable proliferation of the mutp53-expressing cells. Li et al. [74] also reported that another HDACi, vorinostat/SAHA, depletes multiple mutp53 (p53L194F, p53R273H, p53R280K) without altering wtp53 levels. Intriguingly, SAHA-induced degradation of mutp53 is mediated by MDM2 and CHIP E3 ligases following disruption of a complex of HSP90, mutp53, and a positive HSP90 regulator, HDAC6. Furthermore, SAHA preferentially induces cytotoxicity in mutp53-expressing cancer cells, which is synergistic with 17AAG, and depletion of mutp53 by SAHA sensitizes MDA-MB-231 and T47D cells to camptothecin [102]. However, Yan et al. [103] showed that inhibition of HDAC8, a class I HDAC, by NaB and SAHA results in reduced transcription of both wtp53 and mutp53 in HCT116 (p53wt), HaCaT (p53H179Y/R282W), SW480 (p53R273H/P309S), and MIA PaCa-2 (p53R248W) cells. These results may suggest that SAHA reduces mutp53 levels by controlling both transcription and protein stability of mutp53 by acting on different types of HDAC. Recently, Foggetti et al. [104] reported that SAHA induces degradation of mutp53 (p53R280K and p53S241F in MDA-MB-231 and DLD1, respectively) in an autophagy-dependent manner since autophagy inhibition stabilizes mutp53. Thus, there may be multiple mechanisms behind the reduction in mutp53 protein levels by vorinostat/Zolinza/SAHA.

A group from the MD Anderson Cancer Center conducted two phase 1 clinical trials investigating the combinatory effects of vorinostat and MLN9708 (ixazomib, a proteasome inhibitor to induce autophagy) or pazopanib (Votrient, a tyrosine kinase inhibitor that inhibits angiogenesis) in patients with advanced p53-mutated malignancies (NCT02042989, NCT01339871) [28,29]. Patients treated with the combination of pazopanib and vorinostat had clinical benefits with extended progression-free survival (PFS) compared with limited effects in those treated with ixazomib and vorinostat. However, the frequent use of vorinostat caused adverse events including anemia and thrombocytopenia, resulting in dose reduction and patient withdrawal [28]. Moreover, neither study confirmed a reduction in mutp53 levels in tumors. Future clinical trials are needed to examine the correlation between the reduction in mutp53 levels in tumors and clinical outcomes.

### 2.3. Induction of p53 Synthetic Lethality or Targeting Vulnerabilities Imposed by p53 Mutations or Deletions

Synthetic lethality is a fatal phenomenon caused by the simultaneous occurrence of multiple genetic events, while a single event is tolerable [105]. Synthetic lethality can also be observed when a protein or a pathway that plays a crucial role in the survival of cells with a specific gene mutation is inhibited. Thus, synthetic lethality can be induced by targeting vulnerabilities imposed by cancer-specific alterations in cells, which does not cause lethality in non-tumor cells, thereby resulting in minimal side effects and efficient tumor suppression. Some synthetic lethal partners with p53 deletions or mutations have been reported (e.g., Wee1, PKC, PLK1, PARP), many of which play roles in G2 and M cell cycle checkpoints [105,106,107,108]. Considering that p53’s major function is to regulate the G1 cell cycle checkpoint, impairing other cell cycle checkpoints (G2, M) that are crucial for maintaining faithful DNA replication and genomic or chromosome integrity can be deleterious to the cells [17,106,107,109,110,111,112]. Currently, only adavosertib (AZD1775/MK-1775), a Wee1 inhibitor, is in clinical trials to examine whether it efficiently suppresses the progression of tumors with p53 deletions or mutations (p53 deficiency).

#### 2.3.1. p53 Synthetic Lethality Induced by a Wee1 Inhibitor, Adavosertib (AZD1775/MK-1775)

Wee1 protein kinase inhibits Cdk1 and 2 that promote S and G2/M cell cycle progression, and hence Wee1 inhibitors prevent the activation of the G2/M cell cycle checkpoint. Indeed, a Wee1 inhibitor, PD0166285, which inhibits phosphorylation of Cdc2 and attenuates the G2/M checkpoint, sensitizes p53-deficient E6-overexpressing PA-1 cells to γ-irradiation, while it shows little effect on p53-proficient PA-1 cells [113]. Another Wee1 inhibitor, adavosertib (AZD1775/MK-1775), also enhances the cytotoxic effects of several DNA-damaging agents (5-FU, pemetrexed, doxorubicin, camptothecin, mitomycin C) specifically in p53-mutated colorectal cancer cells, while it shows little effect on 5-FU-induced cytotoxicity in wtp53 colon cancer cells [114]. Thus, Wee1 inhibitors can be used to cause p53 synthetic lethality. It should be noted that PD0166285 can still induce cell death at a high concentration in wtp53 cancer cell lines [115], while adavosertib (AZD1775/MK-1775) also induces cell death in sarcoma cells regardless of the p53 status [116,117]. Thus, the efficacy of Wee1 inhibitors to induce efficient p53 synthetic lethality may be dependent on cellular context or tissue type as well as on specific genotoxic stress.

Adavosertib/AZD1775/MK-1775 has been tested in a total of 59 clinical trials. Among these, nine phase 2 trials have been performed with stratification of p53 status in cancers, including ovarian cancer (NCT01164995, NCT01357161, NCT02272790), relapsed SCLC (NCT02688907, NCT02593019), untreated non-small cell lung cancer (NSCLC, NCT02087241), NSCLC (NCT02087176), recurrent uterine serous carcinoma (NCT03668340), and colorectal carcinoma (FOCUS4-C). Specifically, in the NCT01164995 trial, adavosertib/AZD1775 enhanced carboplatin efficacy in p53-mutated ovarian cancer with an overall response rate of 43% [30]. Additionally, in the NCT01357161 trial examining whether adavosertib/AZD1775 could enhance the treatment efficacy of carboplatin/paclitaxel chemotherapy in women with p53-mutated, platinum-sensitive ovarian cancer [31], a modest clinical benefit of adavosertib/AZD1775 was observed by improving PFS (7.9 months vs. 7.3 months; hazard ratio: HR, 0.63). Moreover, in the NCT02272790 trial investigating the efficacy and safety of adavosertib/AZD1775 in combination with commonly used chemotherapy agents (gemcitabine, paclitaxel, carboplatin, or pegylated liposomal doxorubicin) for primary platinum-resistant ovarian cancer, adavosertib/AZD1775 showed some promising outcomes in combination with carboplatin [32]. However, this combination therapy causes more frequent hematologic toxicity than carboplatin monotherapy. Thus, future studies optimizing the treatment dose and schedule are required for the adavosertib/AZD1775 and carboplatin combination.

Additionally, in the FOCUS4-C trial, Seligmann et al. [33] reported that adavosertib/AZD1775 is well tolerated with improved PFS in RAS- and p53-mutated metastatic colorectal cancer compared with active monitoring (3.61 v 1.87 months; HR, 0.3). Furthermore, the NCT03668340 trial tested the safety and efficacy of adavosertib/AZD1775 in recurrent uterine serous carcinoma, in which over 90% of cases have p53 mutations [34]. In this trial, of the 32 tumors examined, all had p53 mutations. Other alternations included mutations in CCNE1 (31%), KRAS (19%), and PIK3CA (41%), as well as gene amplification of Myc (47%) and ERBB2 (19%). Adavosertib/AZD1775 monotherapy demonstrated encouraging and significant activity, with an overall response rate (ORR) of 29.4% and a PFS rate of 6 months. However, this study also suggested that p53 deficiency alone is not sufficient to sensitize tumors to Wee1 inhibition and that some intrinsic stress, including replication stress by CCNE1 mutations or MYC amplifications, may be required for efficient induction of cytotoxicity by adavosertib/AZD1775 Wee1 inhibitor [34]. Additional clinical trials, including NCT02688907, NCT02593019, NCT02087241, and NCT02087176 for SCLC and NSCLC, have been performed; however, the outcomes of these trials are not reported.

Other drugs known to cause p53 synthetic lethality include UCN-01 (PKC inhibitor) and BI-2536 (PLK1 inhibitor). These drugs have been tested in phase 2 clinical trials, including UCN-01 for leukemia, lymphoma, SCLC, metastatic melanoma, metastatic kidney cancer, metastatic pancreatic cancer, fallopian tube cancer, ovarian cancer, and primary peritoneal cavity cancer, as well as BI-2536 for small cell lung cancer, advanced or metastatic NSCLC, refractory or relapsed acute myeloid leukemia, advanced unresectable pancreatic cancer, prostate cancer, and recurrent or metastatic solid tumors. However, none of these trials stratified the p53 status in tumors.

#### 2.3.2. Targeting the Reverse Transcriptase Activity of LINE-1 Enhanced by p53 Deficiency as a Vulnerability

LINE-1 (Long Interspersed Element 1) is a family of retrotransposon elements present in the human genome, consisting of ~15% of the genome [118]. Active LINE-1 elements replicate themselves and can be inserted into new genomic loci, leading to enhanced genomic instability and cancer progression [118,119]. In somatic tissues, LINE-1 is suppressed via DNA methylation, histone H3K9 methylation, and RNA silencing [120], which makes LINE-1-encoded protein ORF1p and PRF2p undetectable. However, LINE-1 is derepressed in many human cancers [121]. Hypomethylation of LINE-1 (derepressed LINE-1) is correlated with poor patient outcomes in gastrointestinal cancers [122,123], oropharyngeal squamous cell carcinoma [124], and hepatocellular carcinoma [125]. Accumulating reports suggest that loss of wtp53 function or p53 mutations are associated with LINE-1 expression, while wtp53 suppresses LINE-1 expression by binding to the L1 internal RNA polymerase II promoter within the 5′UTR [118,126,127]. These findings suggest that derepressed LINE-1 in p53-deficient cancers greatly contributes to genomic instability and malignant progression. Indeed, inhibition of the reverse transcriptase (RT) activity encoded in LINE-1 ORF2p by an FDA-approved non-nucleoside RT inhibitor of HIV-1, efavirenz, results in reduced proliferation in breast cancer cells [128] and pancreatic cancer cells [129]. Consistently, efavirenz inhibits xenograft growth of PC3 cells in nude mice [130,131]. Thus, RT activity in cancer cells, induced by LINE-1, can be a cancer therapeutic target. Indeed, efavirenz has been in clinical trials for multiple types of cancer including metastatic prostate cancer (NCT00964002), metastatic pancreatic cancer (NCT00964171) [132], and solid tumors or non-Hodgkin lymphoma (NCT01878890), although the clinical benefits have not yet been reported. Moreover, none of these trials stratified the p53 status.

Lamivudine (3TC/Epivir/Zeffix/DELSTRIGO) is an FDA-approved synthetic nucleoside RT inhibitor used for the treatment of HIV and HBV, and it specifically reduces human LINE-1 retrotransposition [133]. Recently, Rajurkar et al. [35] reported that lamivudine inhibits the migratory potential, anchorage-independent growth, and growth of tumor xenografts more efficiently in mutp53-expressing colorectal cancer cell lines (DLD1, HCT15, C2BBe1, LS123, SW620) than in cells with wtp53 (HCT8, HCT116, LOVO). They also found that LINE-1 repeat elements are more robustly enriched in wtp53 cells compared with those in mutp53 cells, while baseline LINE-1 RNA expression and the major active human LINE-1 retrotransposon are significantly higher in mutp53 cells than in wtp53 cells. This finding is consistent with the observation that p53 deficiency induces human LINE-1 expression [126].

Based on these findings, Rajurkar et al. [35] have initiated a phase 2 clinical trial of single-agent lamivudine in patients with mutp53-expressing chemo-refractory metastatic colorectal cancers (NCT03144804). Of 32 patients enrolled in this study, stable disease (SD) has been seen in 8 patients on single-agent lamivudine. Analyses of baseline LINE-1 ORF1p protein levels in pretreatment tumor biopsy and serum specimens from patients reveal that the ORF1p levels are significantly higher in the progressive disease (PD) group than in the SD group, suggesting that a higher dose of lamivudine may be required for inhibiting the activity of LINE-1 in the PD group. Nonetheless, their studies encourage the use of lamivudine to inhibit the LINE-1 activity provoked by p53 mutations or deletions that is likely crucial for cancer progression.

#### 2.3.3. Targeting YPA/TAZ Activity Enhanced by GOF mutp53 as a Vulnerability

The Hippo signaling pathway regulates various cellular and tissue homeostatic processes, such as cell survival, proliferation, differentiation, cell regeneration, and organ size, while its dysregulation significantly contributes to malignant progression and drug resistance [134]. YAP (Yes-associated protein) and TAZ (transcriptional co-activator with PDZ-binding motif) are the two key downstream effectors of this pathway, and YAP/TAZ are frequently overactivated in multiple types of human cancers [135,136,137,138]. Thus, controlling YAP/TAZ activity is crucial for cancer therapy.

Increasing evidence indicates that GOF mutp53 functionally enhances the YAP/TAZ activity, which greatly contributes to cancer progression. For example, several DNA contact and structural GOF p53 mutants interact with YAP to form a complex with the oncogenic transcription factor NF-Y and enhance the transcriptional activity [139]. In addition, mutp53 binds to and activates SREBP2, a key transcription factor controlling the expression of enzymes in the mevalonate pathway, leading to increased prenylation of RhoA, inhibited YAP phosphorylation by LATS1/2, and enhanced nuclear translocation and activation of YAP/TAZ [140]. Thus, enhanced YAP/TAZ activity by mutp53 through activation of the mevalonate pathway may play a crucial role in malignant progression of mutp53-expressing cancer cells. Since inhibition of the mevalonate pathway results in reduced protein levels of mutp53 as well [14,91], blockage of this pathway may result in inhibition of both activities of mutp53 and YAP/TAZ.

There are FDA-approved drugs that inhibit the mevalonate pathway. One is the class of cholesterol-lowering drugs called statins which inhibit HMG-CoA reductase, the rate-limiting step of this pathway. The other is zoledronic acid (Reclast/Zometa), one of the most frequently used bisphosphonates that inhibit osteoclast activity and so are used to treat hypercalcemia, osteoporosis, Paget’s disease, multiple myeloma, and cancers with bone metastasis [141,142]. Indeed, Sorrentino et al. [143] showed that cerivastatin or zoledronic acid each inhibits nuclear localization and activity of YAP/TAZ in multiple cancer types. Göbel et al. [144] also reported that a combination of statins (simvastatin, rosuvastatin, atorvastatin) with zoledronic acid has a cooperative effect on inducing apoptosis and cytotoxicity in breast and prostate cancer cell lines. Similar cooperative effects of simvastatin and zoledronic acid have been observed in multiple myeloma cell lines [145]. Thus, the combination of two inhibitors of the mevalonate pathway may allow reducing the doses of these drugs to effectively inhibit tumor progression. However, neither study addresses whether mutp53-expressing cells show higher sensitivity to the mevalonate pathway blockade by this drug combination compared with cells with p53 null or wtp53. Moreover, it remains unclear whether observed anti-tumor effects by the mevalonate pathway blockade are dependent on the inhibition of YAP/TAZ and/or mutp53 GOF activities.

A phase 2 clinical trial (NCT03358017) is underway to examine the effects of the combination of atorvastatin and zoledronic acid on the standard anthracyclines/taxanes-based neoadjuvant chemotherapy in triple-negative breast cancer. This trial not only investigates changes in the YAP/TAZ activity by the treatments, but also assesses the anti-tumor effects of the mevalonate pathway blockade associated with neoadjuvant standard chemotherapy by stratifying p53 protein levels (low ≈ wtp53 vs. high ≈ mutp53). Together, the use of FDA-approved drugs that block the mevalonate pathway may inhibit cancer progression by targeting the activities of two oncogenic factors, mutp53 and YAP/TAZ.

## 3. Discussion

The majority of p53 mutations are missense mutations; however, it remains unclear whether some mutants behave similarly, or each mutant has a different biological character. Missense mutp53 is roughly classified into two categories based on recognition by p53 conformation-specific antibodies under non-denaturing conditions: one is the DNA contact type that retains a relatively intact p53 structure, and the other is the conformational, structural, or misfolded type with robust p53 structural changes. Both mutant types generally lose the p53’s DNA-binding activity. However, these mutant types have different thermodynamic stability of the core DNA-binding domain [146,147]. Hence, drugs that work for one group of mutp53 may not work for the other group. Indeed, statins deplete mainly conformational mutp53 through regulation of binding between conformational mutp53 and DNAJA1, a member of HSP40 [14,148], whereas HSP90 inhibitors appear to deplete both DNA contact and conformational types [13,149]. In addition, APR-246 can reactivate both types of mutp53 [12], whereas ATO reactivates mainly structural or conformational mutp53 [58]. It is important to be able to determine whether these drugs show mutant type-specific activities to reactivate p53 and inhibit cancer progression in clinics consistent with the results in tissue culture and mouse models.

Increasing evidence indicates that even wtp53 sometimes acts like mutp53 under specific conditions, referred to as “pseudo-mutp53”. This finding is detected in cancer-associated fibroblasts (CAFs), in the absence of specific molecular chaperones, and in a subpopulation of pre-leukemic hematopoietic stem or progenitor cells with mutations in DNMT3A (DNA methyltransferase 3 alpha) [150,151,152,153,154]. It would be interesting to examine whether mutp53-reactivating drugs could restore wtp53 activity in cells with pseudo-mutp53.

Two p53 reactivators, APR-246 and ATO, are in clinical trials. Although both stabilize wtp53 conformation and reactive p53 in tissue culture and mouse models, it is yet unclear if p53 reactivation by these drugs is sufficient to inhibit cancer progression as a single agent in human clinical trials. Indeed, the experimental and clinical outcomes of APR-246 are varied [23,24,25,26]. This variability may be due to the fact that only a small portion of mutp53 is reactivated, which can still be inactivated by the DN activity of the remaining non-reactivated mutp53. Additionally, it is unclear how long the restored p53 remains active and whether it is degraded by MDM2 or other ubiquitin ligases. A combination of these p53 reactivators with p53-activating chemotherapy agents, including MDM2 inhibitors, would help maximize the efficacy of the restored p53 activity. Moreover, it needs to be carefully determined whether these drugs could have any ability to stabilize wtp53 and whether they cause adverse effects in humans, especially when combined with other chemotherapies. Additionally, ATO is suggested to induce proteasome-dependent degradation of mutp53 [57,97]. It remains unclear whether ATO can simultaneously induce mutp53 degradation and reactivation or whether the degradation is followed by reactivation.

Depleters of mutp53 include the HSP90 inhibitors and statins mentioned above. HSP90 inhibitors cause MDM2- and CHIP-mediated degradation of both DNA contact and conformational mutp53, whereas statins induce CHIP-mediated degradation of mainly conformational mutp53 [13,149]. Both drugs capitalize upon the addiction of cancer cells to mutp53, since mutp53 is necessary for mutp53-expressing cancers to grow and survive. However, several questions should be clarified in the future, including what the exact mechanisms of tumor suppression by mutp53 depletion are, what the cellular context is that efficiently induces the tumor inhibitory effects of these drugs, which p53 mutants respond to these drugs, and how much levels of mutp53 need to be depleted to inhibit cancer progression.

Drugs that induce p53 synthetic lethality often inhibit proteins or pathways involved in G2 or M (mitotic) cell cycle checkpoints [17,106,110,111,112,155]. However, simply inhibiting proteins or pathways involved in these checkpoints may not be sufficient to efficiently induce tumor suppression. Indeed, genetic knockdown or knockout of ATM (ataxia-telangiectasia mutated) or ATR (ataxia telangiectasia and Rad3-related) itself does not cause lethality in p53-deficient cells [156,157]. In addition, many compounds targeting these checkpoints require a combination of DNA-damaging agents to induce p53 synthetic lethality, which is often specific to cancer types or cellular contexts [16,18,30,113,117,158,159,160,161,162,163]. For example, the p53 synthetic lethal effects of an inhibitor of Wee1 G2 checkpoint kinase (AZD1775) are observed mainly in epithelial cancer cells, and it can inhibit tumor growth of sarcoma cells irrespective of the p53 status [117,159]. To date, none of the drugs has yet been proven to induce p53 synthetic lethality effectively in clinical trials. Thus, the exact mechanisms and cellular contexts for inducing p53 synthetic lethality should be determined to identify efficient strategies that induce cell death specifically in p53-deficient cancer cells.

## 4. Conclusions

Mutations in p53 are cancer-specific and, hence, are ideal molecular targets for targeted cancer therapy that is expected to cause minimum side effects. Several drugs have been developed to target p53 mutations. Although biological effects using cell culture and mouse models have been demonstrated, the clinical safety and efficacy of the majority of drugs targeting p53 mutations need to be determined. To improve the efficacy of mutp53-targeted therapy and reduce side effects, the exact mechanisms of action and efficient strategies to induce cell death specifically in p53-deleted or -mutated cells by these drugs need to be elucidated.

## Figures and Tables

**Table 1:** Summary of drugs targeting p53 mutations.

Drug	Chemical Structure	Action on p53	Trial Identifier	Cancer Type	Reference	Brief Summary/Current Status
APR-246 (eprenetapopt, PRIMA-1^MET^)	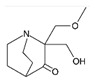	Mutp53 reactivation	NCT04383938	Advanced solid tumor (bladder, gastric, NSCLC, urothelial)	[23]	Well tolerated for the combination with pembrolizumab
			NCT03072043	MDS/oligoblastic AML	[24]	Favorable outcomes with response rates for MDS (73%) and oligoblastic AML (64%)
			NCT03588078	AML/MDS	[25]	Favorable outcomes with response rates for MDS (62%) and AML (33%)
			NCT03931291	AML/MDS in post-HCT maintenance therapy	[26]	Improved RFS
			NCT03745716	MDS	NA	NR, trial completed
			NCT02098343	Platinum-sensitive recurrent HGSOC	NA	NR, trial completed
			NCT03268382	Platinum-resistant recurrent HGSOC	NA	NR, trial completed
			NCT04214860	Myeloid malignancies	NA	NR, trial completed
PEITC (phenethyl isothiocyanate)	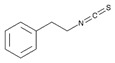	Mutp53 reactivation	NCT01790204	Oral cancer	NA	NR, trial completed
ATO (arsenic trioxide/Trisenox)	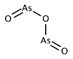	Mutp53 reactivation	NCT03855371	AML/MDS	NA	NR, recruiting patients
			NCT04869475	Refractory solid tumors	NA	NR, recruiting patients
			NCT04489706	Recurrent and metastatic ovarian and endometrial cancer	NA	NR, recruiting patients
			NCT04695223	Refractory solid tumors	NA	NR, recruiting patients
HSP90 inhibitor (ganetespib/STA-9090)	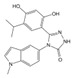	Mutp53 degradation	NCT02012192	High-grade platinum-resistant ovarian cancer	[27]	Confirm safe use of the combination
Atorvastatin	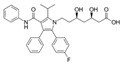	Mutp53 degradation	NCT04767984	Longstanding ulcerative colitis	NA	NR, recruiting patients
			NCT03560882	Solid tumor and relapsed AML	NA	NR, recruiting patients
ATO/Trisenox	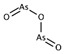	Mutp53 degradation	NCT03381781	AML	NA	NR, not recruiting yet
			NCT03377725	MDS	NA	NR, not recruiting yet
Vorinostat/Zolinza/SAHA	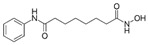	Mutp53 degradation	NCT02042989	Advanced malignancies	[28]	Limited effects
			NCT01339871	Advanced malignancies	[29]	Extended PFS
Wee1 inhibitor (adavosertib/AZD1775/MK-1775)	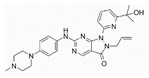	Synthetic lethality to p53	NCT01164995	Refractory and resistant ovarian cancer	[30]	Enhance carboplatin efficacy
			NCT01357161	Platinum-sensitive ovarian tumors	[31]	Modest clinical benefit with improved PFS
			NCT02272790	Platinum-resistant ovarian cancer	[32]	Some promising outcomes with carboplatin
			FOCUS4-C	Metastatic colorectal cancer with RAS	[33]	Improved PFS
			NCT03668340	Recurrent uterine serous carcinoma	[34]	Significant activity (but p53 deficiency alone is not sufficient)
			NCT02688907	Relapsed SCLC with CDKN2A	NA	NR, trial terminated
			NCT02593019	Relapsed SCLC with CDKN2A	NA	NR, trial completed
			NCT02087241	Untreated stage IV NSCLC	NA	NR, trial terminated
			NCT02087176	NSCLC	NA	NR, trial terminated
Lamivudine (3TC/Epivir/Zeffix/DELSTRIGO)	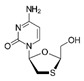	Inhibition of LINE-1 upregulated by p53 loss	NCT03144804	Metastatic colorectal cancer	[35]	SD in 8 out of 32 cases
Zoledronic acid (ZA/Reclast/Zometa) and atorvastatin	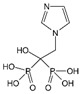 ZA	Inhibition of YPA/TAZ activity enhanced by mutp53	NCT03358017	Triple negative breast cancer	NA	NR, recruiting patients

AML: acute myeloid leukemia; MDS: myelodysplastic syndromes; HCT: hematopoietic stem-cell transplant; HGSOC: high grade serous ovarian cancer; SCLC: small cell lung cancer; NSCLC: non-small cell lung cancer; NA: not applicable; RFS: relapse-free survival; NR: not reported; PFS: progression-free survival; SD: stable disease.

## Data Availability

Not applicable.
